# Clinical analysis of 50 patients with heterotopic pregnancy after ovulation induction or embryo transfer

**DOI:** 10.1186/s40001-018-0316-y

**Published:** 2018-04-16

**Authors:** Zaigui Wu, Xinmei Zhang, Ping Xu, Xiufeng Huang

**Affiliations:** Women’s Hospital, School of Medicine, Zhejiang University, Hangzhou, China

**Keywords:** Heterotopic pregnancy, Ectopic pregnancy, Controlled ovarian hyperstimulation

## Abstract

**Objectives:**

The purpose of this study was to evaluate the clinical characteristics, prenatal diagnosis, and management of patients with heterotopic pregnancy after ovulation induction or embryo transfer.

**Methods:**

This was a retrospective study of fifty cases with heterotopic pregnancy, in which the fertilization way, type and number of embryo transferred, gestational age, clinical presentation and outcome of intrauterine, ultrasound presentation and site of ectopic pregnancy, bilateral fallopian tube and treatment were evaluated.

**Results:**

Six patients had spontaneous pregnancy and two had artificial insemination after ovulation induction. Sixteen had fresh and nineteen had frozen embryo transfer with seven patients unrecorded its embryo type and number. The average days from transplantation (or intercourse/insemination) to diagnosing heterotopic pregnancy was thirty-seven with the earliest eighteen and the latest more than 70 days. Although the most common presentation was vaginal bleeding or abdominal pain, more than 21% was found by ultrasound and rare individuals even presented with gastrointestinal symptom which may imply ruptured EP and hemorrhagic shock. Giving proper treatment (surgery or local drug injection), the majority of them had a successful intrauterine pregnancy with only seven miscarried.

**Conclusions:**

Ovulation induction or embryo transfer increased the risk of HP greatly and clinician should raise high suspicious during the whole first trimester. Although the most ectopic site was ampullary, other sites such as cornual, cervical, abdominal especially interstitial or tubal stumps should also be assessed by ultrasound even in patients with bilateral salpingectomy or tubal obstructed. Repeated ultrasound tests 2 weeks after the diagnosis of intrauterine pregnancy with heart beating was very necessary to find the missed ones in suspicious patients. Compared with surgery, embryo suction with or without proper local drug injection would be more advisable for patients with cervical, cornual, or interstitial pregnancy in order to reserve the intrauterine pregnancy.

## Background

Heterotopic pregnancy (HP), first reported in 1708, was defined as the co-incidence of intrauterine and ectopic pregnancy. That is, the embryos implanted at two or more different sites simultaneously. In natural cycles, usually only one follicle developed and ovulated, the spontaneous incidence of HP was extremely low (one in 30,000 pregnancies) [1]. Due to the extended use of assisted reproductive technologies (ARTs), the incidence of HP had been reported to be up to 1–11% [2]. Therefore, early diagnosis and proper treatments were very important for them to remove the ectopic pregnancy (EP) while reserve the intrauterine pregnancy (IUP).

In clinic, it was often difficult to diagnose the HP as early as possible because large amount of them were asymptomatic or just masked by enlarged ovaries after ovulation induction [3, 4]. The most common EP site was fallopian tubal, but other sites such as cornual, cervical, abdominal, interstitial, or tubal stumps pregnancy all had been reported in the literature [5] and our paper. Thus, the clinician and sonographer should raise high suspicious of HP with patients transplanting multiple embryos or having ovulation induction.

Once HP was diagnosed, the optimal solution should be carried out. The choice of therapeutic approach relied on many factors, of which the vital one is to preserve the coexisting IUP as possible. First, systematic methotrexate or embolization was not suitable for HP patients unless the IUP aborted. Second, the traditional salpingectomy may be still practicable to HP patients with tubal pregnancy, but for special types such as cornual and interstitial pregnancy the conventional management with salpingectomy and cornual resection should no longer be the first choice. The alternative method would be embryo suction combined with local injection of MTX or 10% KCL [6]. It, to a certain extent, could relieve the risk of uterine rupture during the subsequent pregnancy.

The purpose of this paper was to review all patients who had HP at our center from 2007 to 2015 to make the clinician impressed on the diagnosis and treatment of HP.

### Patients and methods

This retrospective study included all the patients diagnosed with HP from January 2007 to September 2015 at the study center. In our center, the first ultrasound test was carried out 4–5 weeks after ET in general and a repeated test was also performed 2 weeks after the diagnosed of an IUP with heart beaten. If patients had vaginal bleeding or abdominal pain ultrasound test would be taken at any time. Fertilization way, gestational age at diagnosis, the ultrasound characteristics of IUP and EP, clinical symptoms, bilateral fallopian tubes, EP site, and treatments were recorded and analyzed; the results are described in Tables 1, 2, 3, 4, and 5.

**Table 1:** Clinical data of eight patients of tubal HP with heart beaten of extrauterine pregnancy

Case	Fertilization way (embryo type and number if ET)	Gestational age (days after ET or intercourse)	Clinical symptoms	Presentation and outcome of IUP	Extrauterine pregnancy presentation	Bilateral fallopian tubes	EP site + treatment
1	IVF-ET (fresh 2)	26	Vaginal bleeding 5 days	CRL 0.4 cm, heart beaten	Left adnexal mass 5 cm, CRL 0.3 cm with heart beaten	Chronic inflammation by HSG	Ampullary + laparoscopy salpingectomy
2	IVF-ET (frozen 3)	39	Vaginal bleeding 18 days	CRL 0.8 cm, heart beaten	Right adnexal mass 3 cm, CRL 1.0 cm with heart beaten	Bilateral unobstructed by HSG	Ampullary + laparoscopy salpingectomy
3	IVF-ET (fresh 2)	26	Vaginal bleeding 10 days	CRL 0.2 cm, heart beaten	Left adnexal mass, CRL 0.2 cm with week heart beaten	Right salpingectomy for EP	Ampullary + laparoscopy salpingectomy
4	IVF-ET (frozen 2)	52	Lower abdominal pain 2 days	CRL 3.5 cm, heart beaten	Right adnexal mass 5 cm CRL 2.7 cm, with heart beaten	Bilateral tubal inflammation	Ampullary + laparotomy salpingectomy
5	IVF-ET (frozen 3)	23	Vaginal bleeding 6 days and abdominal pain 1 day	CRL 0.4 cm, heart beaten	Right adnexal mass 4 cm, CRL 0.4 cm with heart beaten	Bilateral unobstructed by HSG	Ampullary + laparotomy salpingectomy
6	IVF-ET (unknown)	32	Bleeding and pain 4 days	CRL 0.7 cm, heart beaten	Right adnexal mass, CRL 0.5 and 0.4 cm both with heart beaten	Left salpingectomy and right obstructed by HSG	Ampullary + laparoscopy salpingectomy
7	IVF-ET (fresh 2)	54	Asymptomatic, routine ultrasound test found	CRL 3.2 CM, heart beaten	Left adnexal mass 7 cm CRL 1.8 cm with heart beaten	Left salpingostomy and right salpingectomy both for EP	Ampullary + laparotomy salpingectomy
8	Ovulation induction + spontaneous	38	Asymptomatic, routine ultrasound test found	CRL 1.9 cm, heart beaten	Right adnexal mass 3 cm, yolk sac without embryonic pole	Bilateral tubal unobstructed by laparoscopy hydrotubation	Ampullary + laparotomy salpingectomy

**Table 2:** Clinical data of fifteen patients of tubal HP with adnexal mass

Case	Fertilization way (embryo type and number if ET)	Gestational age (days after ET or intercourse)	Clinical symptoms	Presentation and outcome of IUP	Extrauterine pregnancy presentation	Bilateral fallopian tubes	EP site + treatment
9	IVF-ET (fresh 2)	30	Vaginal bleeding 12 days	CRL 0.9 cm, heart beaten	Right adnexal mass 5 cm	Left salpingostomy for hydrops	Ampullary + laparotomy bilateral salpingectomy
10	IVF-ET (fresh 3)	29	Vaginal bleeding 2 days	CRL 0.4 cm, heart beaten	Left adnexal mass 2 cm	No abnormalities by laparoscope	Ampullary + laparotomy salpingectomy
11	IVF-ET (frozen 2)	38	Vaginal bleeding 3 days	CRL 0.7 cm, heart beaten	Left adnexal mass 3.5 cm	Left chronic inflammation while right normal by HSG	Tubal + laparotomy salpingectomy
12	Ovulation induction + IUI	52	Vaginal bleeding 30 days	CRL 2.2, 2.4 cm, both with heart beaten	Left adnexal mass 7 cm	Bilateral tubal inflammation but not obstructed by HSG	Tubal + laparoscopy salpingectomy
13	IVF-ET (unknown)	35	Vaginal bleeding	CRL 0.2 cm, week heart beaten → live birth	Right adnexal mass 3 cm	Secondary infertility	Tubal + laparotomy salpingectomy
14	IVF-ET (fresh 2)	21	Right hypogastrium dull pain 1 day	CRL 0.5 cm, heart beaten	Right adnexal mass 6 cm	Left salpingostomy for EP	Ampullary + laparotomy bilateral salpingectomy
15	IVF-ET (frozen 2)	35	Lower abdominal bulge 12 h	CRL 1.1 cm, heart beaten	Right adnexal mass 7 cm	Bilateral tubal repair surgery for EP	Tubal + laparotomy salpingectomy
16	IVF-ET (frozen 2)	31	Lower abdominal pain 1 day	CRL 1.5 cm, heart beaten	Left adnexal mass 3 cm	Right salpingostomy for EP and bilateral salpingostomy 1 year later	Tubal + laparotomy salpingectomy and contralateral tubal cutoff
17	IVF-ET (frozen 2)	53	Left abdominal pain 1 h	CRL 3.6 cm, heart beaten	Left fundus mass 4 cm	Right salpingectomy for EP and left tubal obstructed	Tubal + laparoscopy salpingectomy
18	Ovulation induction + IUI	30	Vaginal bleeding 2 times and abdominal pain 1 day	Yolk sac without embryonic pole → live birth	Left adnexal mass 6.5 cm	Bilateral unobstructed by HSG	Ampullary + laparotomy salpingectomy for ruption abdominal haematocele 800 ml
19	Ovulation induction + spontaneous	33	Vaginal bleeding 1 day and abdominal pain 6 h	CRL 0.2 cm, heart beaten	Right adnexal mass 6.5 cm	Bilateral unobstructed by HSG	Ampullary + laparoscopy salpingectomy
20	Ovulation induction + spontaneous	40	Asymptomatic, routine ultrasound test found	CRL 0.7 cm, heart beaten	Right adnexal mass 3 cm	Left salpingectomy for EP	Tubal ampullary + laparoscopy salpingectomy
21	IVF-ET (frozen 3)	30	Asymptomatic, routine ultrasound test found	Both CRL 2.1 cm, heart beaten	Right adnexal mass 3 cm	History of pelvic surgery	Ampullary + laparotomy salpingectomy
22	IVF-ET (fresh 2)	28	Asymptomatic, routine ultrasound test found	CRL 0.2 cm, week heart beaten → live birth	Right adnexal mass 4 cm	History of pelvic surgery	Tubal + laparotomy salpingectomy
23	IVF-ET (fresh 2)	22	Haemorrhagic shock	Yolk sac without embryonic pole → live birth	Right adnexal mass 3 cm		Ampullary + laparoscopy salpingectomy

**Table 3:** Clinical data of four patients of tubal HP with the IUP miscarriaged

Case	Fertilization way (embryo type and number if ET)	Gestational age (days after ET or intercourse)	Clinical symptoms	Presentation and outcome of IUP	Extrauterine pregnancy presentation	Bilateral fallopian tubes	EP site + treatment
24	IVF-ET (unknown)	27	Vaginal bleeding 1 day	Abortion	Right adnexal mass 3 cm, CRL 0.2 cm with heart beaten	Chronic inflammation by laparoscope	Tubal + laparoscopy salpingectomy
25	IVF-ET (fresh 2)	32	Vaginal bleeding 7 days	Yolk sac without embryonic pole → abortion	Right adnexal mass, CRL 0.5 cm with heart beaten	Right salpingectomy and left salpingostomy both for EP	Ampullary + laparoscopy salpingectomy
26	IVF-ET (fresh 3)	27	Asymptomatic, routine ultrasound test found	Yolk sac without embryonic pole → missed abortion	Right adnexal mass 2 cm CRL 0.2 cm with heart beaten	Tubal infertility	Tubal + laparoscopy salpingectomy
27	IVF-ET (frozen 2)	58	Nausea and vomiting	CRL 3.2 cm, with heart beaten 3 weeks ago → missed abortion	CRL 2.6 cm without heart beaten hemoperitoneum	Bilateral obstructed by laparoscope	Ampullary + laparotomy salpingectomy, abdominal haematocele 2000 ml

**Table 4:** Clinical data of ninth patients with special site HP treated with suction and/or local injection

Case	Fertilization way (embryo type and number if ET)	Gestational age (days after ET or intercourse)	Clinical symptoms	Presentation and outcome of IUP	Extrauterine pregnancy presentation	Bilateral fallopian tubes	EP site + treatment
28	Ovulation induction + spontaneous	31	Vaginal bleeding 3 times	CRL 1.4 cm, heart beaten	Cervical canal mass CRL 1.2 cm with heart beaten	Bilateral unobstructed by HSG	Cervical + local 10% KCL 10 ml + MTX 40 mg
29	IVF-ET (unknown)	30	Vaginal bleeding 11 days	CRL 0.72 cm, heart beaten	Right cornual mass, CRL 0.61 cm with heart beaten	Double salpingectomy	Interstitial + local MTX 30 mg
30	IVF-ET (unknown)	36	Vaginal bleeding 1 day	CRL 0.9 cm, heart beaten	Right cornual mass 3 cm, CRL 0.75 cm with heart beaten	Unknown	Interstitial + local MTX 30 mg → cornual resection 3 weeks later for mass enlargement
31	IVF-ET (fresh 2)	45	Vaginal bleeding 2 days	CRL 2.45 cm, heart beaten	Right cornual mass 4 cm	Bilateral tubal obstructed by HSG	Interstitial + sunction and local MTX 20 mg
32	IVF-ET (frozen 3)	30	Left abdominal pain 22 days	CRL 0.9 and 0.8 cm, both with heart beaten	Right adnexal mass 2 cm, CRL 0.7 cm with heart beaten	Right salpingectomy for EP	Tubal stumps + sunction and local MTX 20 mg → laparotomy tubal stumps resection for mass ruption
33	IVF-ET (frozen 2)	51	Seldom hypogastrium dull pain 34 days	CRL 3.0 cm, heart beaten	Right adnexal CRL 0.3 cm without heart beaten	Right salpingectomy and left tubal chronic inflammation	Interstitial + sunction and local MTX 20 mg
34	IVF-ET (frozen 3)	33	Asymptomatic, routine ultrasound test found	Both CRL 1.1 cm, heart beaten	Left cornual mass 4 cm, CRL 0.7 cm with heart beaten	Bilateral salpingectomy both for EP	Interstitial + sunction and local MTX 20 mg → cornual resection 2 weeks later for mass ruption
35	IVF-ET (frozen 3)	32	Asymptomatic, routine ultrasound test found	CRL 1.1 cm, heart beaten	Right cornual CRL 0.6 cm with heart beaten	Right salpingectomy and left tubal cutoff for infertility	Cornual EP + sunction by US and local MTX 20 mg
36	IVF-ET (fresh 2)	33	Asymptomatic, routine ultrasound test found	CRL 0.9 cm, heart beaten	Right cornual CRL 0.7 cm with heart beaten	Left salpingectomy for EP	Cornual EP + sunction by US

**Table 5:** Clinical data of fourteen patients with special site HP treated with surgery

Case	Fertilization way (embryo type and number if ET)	Gestational age (days after ET or intercourse)	Clinical symptoms	Presentation and outcome of IUP	Extrauterine pregnancy presentation	Bilateral fallopian tubes	EP site + treatment
37	IVF-ET (fresh 2)	28	Vaginal bleeding 24 days	CRL 0.3 cm, heart beaten	Right adnexal mass 2 cm CRL 0.2 cm without heart beaten	Right salpingectomy and left ligament for EP	Tubal stumps + laparotomy tubal stumps resection
38	IVF-ET (frozen 2)	18	Vaginal bleeding 12 days	Yolk sac without embryonic pole → live birth	Left adnexal mass 7 cm, yolk sac without embryonic pole	Left isthmus ligament	Tubal stumps + laparotomy tubal stumps and part cornual resection
39	IVF-ET (fresh 2)	57	Vaginal bleeding 3 days	CRL 3.7 cm, heart beaten	Left cornual CRL 3.2 cm with heart beaten (3 weeks later)	Bilateral tubal unobstructed by HSG, history of pelvic surgery	Interstitial + laparotomy salpingectomy and part cornual resection
40	IVF-ET (frozen 3)	26	Abdominal pain 3 day	CRL 0.2 cm, heart beaten	Left adnexal mass 4 cm	Bilateral salpingectomy for EP	posterior uterine + laparotomy mass removal, abdominal haematocele 1400 ml
41	IVF-ET (unknown)	> 70	Abdominal pain and vaginal bleeding 12 + days	CRL 4.7 cm, heart beaten	Left fundus mass 5 cm	Left tubal hydrosalpinx	Interstitial + laparotomy salpingectomy and cornual resection
42	IVF-ET (fresh 2)	46	Vaginal bleeding and abdominal distension 6 days	CRL 0.3 cm, live birth→missed abortion	Right adnexal mass 4 cm, CRL1.8 cm without heart beaten	Bilateral salpingectomy	Cornual EP ruption + laparotomy cornual removal,abdominal haematocele 2000 ml
43	IVF-ET (unknown)	22	Vaginal bleeding 11 days and abdominal pain 3 days	Abortion	Mass behind uterus 9 cm	Bilateral unobstructed by laparoscope	Abdominal gestation + laparotomy mass removal
44	IVF-ET (frozen 3)	39	Asymptomatic, routine ultrasound test found	CRL 0.4 cm without heart beaten → missed abortion	Right cornual mass 2.5 cm	Right salpingectomy for EP and left salpingostomy for hydrosalpinx	Interstitial + laparoscopy part cornual resection and local MTX 20 mg
45	Ovulation induction + spontaneous	> 63	Asymptomatic, routine ultrasound test found	CRL 2.6 cm, heart beaten	Right interstitial portion mass 5 cm CRL 2.4 cm, with heart beaten	Left tubal obstructed and right tubal unobstructed by HSG	Interstitial + laparotomy salpingectomy and part cornual resection
46	IVF-ET (frozen 2)	32	Asymptomatic, routine ultrasound test found	CRL 0.6 cm, heart beaten	Left cornual CRL 0.7 cm with heart beaten	Left salpingectomy and right tubal repair surgery	Interstitial + laparotomy part cornual resection
47	IVF-ET (frozen 2)	35	Asymptomatic, routine ultrasound test found	CRL 1.2 cm, heart beaten	Right cornual CRL 0.7 cm with heart beaten	Right salpingectomy and cornual resection both for EP	Cornual–interstitial EP + laparotomy salpingostomy
48	IVF-ET (fresh 2)	36	Asymptomatic, routine ultrasound test found	CRL 1.1 cm, heart beaten	Right cornual CRL 1.1 cm with heart beaten	Bilateral salpingectomy	Cornual ruption + laparotomy cornual resection
49	IVF-ET (frozen 3)	38	Asymptomatic, routine ultrasound test found	CRL 1.2 cm, heart beaten	Right adnexal mass 3 cm	Bilateral tubal obstructed by HSG	Interstitial + laparotomy salpingectomy and cornual resection
50	Ovulation induction + spontaneous	30	Nausea and vomiting	CRL 3.1, 3.2, 4.4 and 1.9 cm all with heart beaten	Right adnexal mass 3 cm	Ovulation induction for PCOS and bilateral tubal unobstructed by HSG	Isthmic EP ruption + laparotomy salpingectomy, abdominal haematocele 2000 ml

## Results

A total of fifty patients were identified with HP (Tables 1, 2, 3, 4 and 5). Coexisting with the IUP, forty-two patients had conceived with IVF-ET, two with intrauterine insemination (IUI) and the other six conceived naturally after ovulation induction. The average time being diagnosed was 37 days after transplantation (or insemination/intercourse) with the shortest 18 days and the longest more than 9 weeks (CRL 4.7 cm). Thirty-two patients had clinical manifestation (fifteen only with vaginal bleeding, eight only with abdominal pain while nine with both), one with hemorrhagic shock due to the EP rupture, another two only with vomiting while the remaining fifteen were asymptomatic.

All patients were diagnosed by ultrasound among whom the visualization of heart activity in both intrauterine and extrauterine gestations was observed in twenty patients. There were twenty-five patients with heart activity seen only in IUP (four had a visible gestational sac in extrauterine gestations while the other twenty-one possessed an adnexal mass). Seven patients were diagnosed with IUP miscarriage (of the extrauterine pregnancy, three with and two without heart activity, two with an adnexal mass) based on the inability to see an embryonic pole or cardiac activity between 6 and 10 weeks. In five patients, transvaginal ultrasound revealed an intrauterine pregnancy with heart beaten appropriate for seven to 8 weeks, gestation while no suggestive findings of an ectopic pregnancy were demonstrated and they were eventually diagnosed with HP two to 3 weeks later.

The most common HP type was IUP complicated with tubal pregnancy including twenty-seven ampullary EP, one isthmic EP, eleven interstitial EP and three tubal stumps pregnancies. Of the rest of eight cases, five were cornual, once was cervical, and two were abdominal. Depending on the location of the ectopic pregnancy, the method of transvaginal ultrasound-guided suction aspiration with or without intracardiac MTX or potassium chloride (KCL) injection had been carried out in five interstitial EP, two cornual EP, one tubal stumps pregnancy, and one cervical EP patients. Due to the rupture of EP, the tubal stumps pregnancy and one interstitial pregnancy patients had the emergency surgery 1 or 2 weeks after the above treatment and another interstitial pregnancy patient had the same experience for enlargement of the adnexal mass 3 weeks later.

As to bilateral fallopian tubes, eleven patients had prior ipsilateral salpingectomy for tubal pregnancy with four interstitial EP, four cornual EP, and three tubal stumps pregnancy this time. Two earlier experienced ipsilateral salpingostomy, and the other five had a history of contralateral tubal pregnancy. A ruptured abdominal pregnancy was observed after bilateral salpingectomy for tubal pregnancy while two interstitial and three ampullary pregnancy patients proved to be the tubal obstruction by hysterosalpingography (HSG) or laparoscopic surgery. Thirteen patients had a history of bilateral salpingitis and the remaining twelve had normal tubes.

Tables 1, 2, 3, 4, and 5 show clinical data of fifty patients with HP in the present study.

## Discussion

It was established that the assisted reproduction techniques (ART) had significantly increased the incidence of HP [7] which could also be showed from our center previous study [5] and this report. According to its definition, we could easily understand ovulation induction or transferring multiple embryo was just the first step to make multi-site implantation possible. There were many other risk factors predisposing the development of HP and we suggested that tubal malfunction worked vitally.

Tubal pregnancies were the most common type of EP with HP as described in our study with twenty-six ampullary in fifty HP patients. The recurrent rate of EP ranges from 15 to 20% in condition to one previous EP treated by linear salpingostomy and it increases to 32% in patients with two [8]. Of the fifty HP patients, fifteen had a history of previous EP with the most three times. The exact pathomechanism was unclear while the latest literature [9, 10] suggested tubal malfunction had a predominant role in the pathogenesis of tubal implantation altering the tubal transport mechanisms and expression of molecules normally inhibiting tubal implantation. It was just the alteration of transport mechanisms and molecules expression that explained why ipsilateral salpingectomy, in clinical, could not totally prevent the recurrent EP but to increase the occurrence of cornual–interstitial pregnancy. In our present study, a total of twenty patients had ipsilateral salpingectomy or even bilateral salpingectomy but this time five had cornual, four had interstitial, and two had tubal stump pregnancy. Moreover, abdominal EP may even be happened on the condition of bilateral salpingectomy which has been reported only four cases with two of ours [11–13]. All together suggested that the risk factors may alter the transport mechanisms and molecules expression systemically. Thus when having ultrasound test, not only the fallopian tubes but also the cervix, previous cesarean scar, cornual region, ovaries, and even abdominal cavity should all be examined carefully regardless of bilateral salpingectomy or tubal obstruction.

As HP was relatively rare and clinical features were nonspecific, the misdiagnosis or delayed diagnosis occurred very frequently [7, 14]. We could see that a great number of patients were asymptomatic (29% in our study and 50% in the literature [15]) or just presented with little vaginal bleeding and/or abdominal pain which could be mistaken for threatened abortion of IUP. Gastrointestinal symptoms such as vomiting or diarrhea can also be seen at times and even some patients experienced shock directly due to the rupture of EP [14]. Careful transvaginal ultrasound evaluation should be performed with these symptomatic patients at any time as the earliest gestational age at ultrasound diagnosis of HP in our center was only 18 days after ET. But for those asymptomatic patients, the first transvaginal ultrasound evaluation can be carried out at 4–5 weeks after ET (or 6–7 gestational weeks) as reported in our study [16]. When the diagnosis of HP was made, a gestational sac was visible in more than half of the extrauterine pregnancies and a heart beaten was even seen in 44% of these patients with the others possessing an adnexal mass. But ten patients (20%) were diagnosed with HP at more than 9 weeks and four of them even had an intrauterine pregnancy appropriate for more than 7 weeks’ gestation while no ultrasonic findings were detected to imply an ectopic pregnancy on her initial ultrasonic testing. Because patients treated with ovulation or ET usually had exact fertilization date, the embryonic development of intrauterine and extrauterine estimated by crown-rump length (CRL) differed no more than 1 week and it was certain that the diagnosis was delayed. As noted previously, above two-thirds patients require two or more ultrasound examinations to establish a diagnosis of HP, and repeated ultrasonic testing 2 weeks after the initial diagnosis of live IUP was very necessary to exclude the diagnosis of HP.

When diagnosed and treated timely and properly, the survival rate of the IUP in heterotopic pregnancies reached to 70% [17]. A large majority of HP (31/50) cases were diagnosed before 35 days after fertilization or ET and only seven patients miscarriaged eventually with the IUP. The treatment options include surgical, medical, and expectant therapy. Seven cases diagnosed with suspicious HP for a adnexal mass less than 4 cm were managed by expectant therapy successfully and they were not reported in our present study. Surgical management was performed in the overwhelming majority of HP cases as reported 78–90.78% in the literature [4, 18] and 81.63% in our report. Regarding ampullary and isthmic EP, salpingectomy via laparoscopy or laparotomy would not increase uterin rupture risk in subsequent pregnancy. But for special local EP such as cornual or interstitial EP and especially cervical pregnancy, surgery would increase the risk of IUP abortion or uterine rupture in future pregnancy. Medical treatment would provide an effective choice for these patients [19]. It consisted of suction aspiration or a combination with injections of MTX, KCL in the gestational sac or fetal heart [20]. The use of MTX was contraindicated previously because of its potential embryotoxicity on the IUP but recent studies have proved its safety and effectiveness with local injection of low-dose [21]. Four interstitial, two cornual, and one tubal stumps pregnancy were managed with sonographically guided suction aspiration and MTX injection alone while a combination of MTX and KCL was applied in the cervical pregnancy patients. Two interstitial and one tubal stumps pregnancy were eventually had surgery due to the rupture of EP in the following-up 1–3 weeks after the procedure. So patients choosing the medical treatment should be informed the risk of EP rupture and repeated ultrasound testing should be followed weekly. Once any clinical suspicion of ectopic pregnancy rupture occurs, a diagnostic laparoscopy or laparotomy must be done immediately.

In conclusion, data from our study with fifty cases showed that any patients concepted by ovulation induction or ET could have the possibility of HP in spite of bilateral salpingectomy or tubal obstruction. Careful and repeated ultrasound testings should be carried out until the diagnosis of HP was excluded during the whole first trimester. Suction and local medical treatment was a feasible option for selected patients with special types of HP especially cornual, interstitial or cervical pregnancy.
